# Traveling Letters

**Published:** 1844-06

**Authors:** 


### THE WESTERN JOURNAL.

New Series.—Vol. I.—No. VI.

LOUISVILLE, JUNE 1, 1844.

### TRAVELING LETTERS FROM THE SENIOR EDITOR.

NO. I.

Mobile, April 23d, 1844.

*To my Colleagues:*

Getlemen:—I have now been in this, the second city of the gulf coast, for nearly a fortnight, and must report myself to you and our readers. You may fancy that I am among French and Spanish creoles, with numerous opportunities of observing the physiology and diseases of the descendants of France and Spain; but such is not the fact, for this is nothing more nor less than a veritable Yankee or New York trading town; the mass of the people being emigrants from the old thirteen, with even a larger population from the states of the north than can be found in Louisville. Of course they have brought with them the temperaments and predispositions of the families from which they were detached; and do not present to the eye of a northern physiologist any thing very new. Howbeit, Mobile is not without a considerable number of French, and a smaller number of Spanish creoles, a numerous mulatto and quadroòn population, who speak the French tongue, and a body of Irish immigrants, much greater than I had expected to see. Apart from the marines, the last make up the majority of the inmates of the two extensive hospitals, of which, before closing, I shall say something.

*Topography of the City.*

The front squares of the city plat were originally a cypress swamp, into which the high tides of the bay were wont to flow. This border is now filled up with sand and built upon. This sand was brought from a plain or terrace in the rear, and the streets which start from the bay rise gradually to an elevation of fifteen or twenty feet. This plateau, naturally covered with pine and oak, stretches to the west five or six miles, when it terminates at the base of another plateau, about 100 feet high, a part of which makes the summer retreat of many families. Out of these wooded sand-hills several copious springs supply the city with very pure and soft water, having a temperature, before it enters the aqueduct, of 68°. Of course it does not get any cooler in making its way into the city, not far below the surface of the ground, in the latitude of 30° 45′; but ice is as "plenty as blackberries," (which by the way, are now on our tables), and so cheap as to be used profusely in all the drinking houses, and by nearly all classes of people. It was long since observed that no body died in Mobile from drinking water when heated in summer; and the reason assigned was the high temperature of the former compared with the well-water of Philadelphia and New York, where such casualties are said to be common. This opinion, however, was killed off by ice, when our medical brethren found that iced water failed to kill any of those laborers who drank it profusely at mid-summer, and there the matter rests, and there I will leave it, and return to Spring Hill. The road to this summer retreat lies through a series of beautiful country seats, much more numerous and elegant than those which embellish our boasted Bardstown-road.

I have brought you back to the pine woods to astound you with a notice of the

*Thundering Spring,*

which has been noised abroad in these parts for the last four years, and to which on Monday last, I made a sort of philosophical pilgrimage, in company with some ladies of taste and observation. You may smile at such companionship in a tour of medical inquiry, but recollect that this was a rural excursion into the midst of natural scenery, and that the eyes and tastes of both sexes are necessary to a full perception of all the aspects which exterior nature presents. The latter part of our trip lay through a lofty forest of long-leaved pine, with but little shrubbery or under-brush, as the fire annually runs over the surface. The spring boils up about fifty feet below the summit level, and flows off into an adjoining woodland swawp or marsh, making one of the sources of "three mile creek;" which is bounded by swamps to its junction with Mobile river, two or three miles above the city. The quantity of water driven up and discharged by the thundering spring, is such as to give it a high rank among the fountains, (I shall not attempt a closer estimate). It boils up in such a manner as to suggest the escape of air, but none is emitted. This boiling is not uniform, but now and then there comes a momentary exacerbation, when the copious but humble *jet d'eau* rises several inches above the surface. A quantity of yellow sand is constantly rising with the strong ascending current, and falling back into the crater, which is an irregular aperture or chasm, in a friable, tertiary sand-stone of a reddish color. When a pole is run into this rocky orifice, it is found to be irregular and several feet in diameter; at the distance of eight or ten feet it is stopped by some obstruction. But I must come to the thundering part of my description. We had stood near the fountain but a moment, when we heard, under our feet, a noise like that of low distant thunder, or the rumbling of a carriage over the pavement of the street when we are seated at the fire-side with the doors and windows closed. It seemed like the sound of a subterranean, and irregularly intermittent cataract. On applying the ear to the trees and saplings which grow around the spot, the same sound was heard still more distinctly, and, moreover, a constant vibration was perceptible. I had not time, by a series of observations, to ascertain the distance from the spring, to which this tremor is propagated; but the owner, Mr. Pierce, who lives on the summit level, about two hundred yards off, assured us that the sound was often heard by his family. He made the discovery of this spring about four years ago, and immediately conceiving that it possessed some extraordinary virtues, determined on making it a watering place for the fashionables of the city, and an asylum from the yellow fever. For aught that can be known, he might have regarded it as the fountain of health and rejuvenescence, which Ponce de Leon sought when he landed on the shores of Florida. Like any other pains-taking and industrious man, he resolved not to rely merely on the fountain's voice to call out the ladies and gentlemen of Mobile, but captured and encaged a rattlesnake, whose *gong* might co-operate with the groanings of the gnomes and nymphs below; but neither the voices calling from this "vasty deep," nor the rattle of the watchman of the woods above, have as yet assembled the expected throng of customers. In fact when tested with reagents, the water is free from every foreign ingredient except a trace of muriate of soda. Its purity indeed, is just as great as that of the neighboring springs, from some of which the city is supplied; but I found its heat 69°, or one degree greater than the other copious fountains round about. Such is the thundering spring, the theory of which I shall leave to yourselves and the geologists, returning to the city to give you a notice of some of its institutions; beginning with

### *The Profession.*

On the whole, I am disposed to believe that Mobile is not quite so much crowded with the messengers of life and death, as most other towns I have visited; but of course I have not become acquainted with the whole, and cannot even state the number. Those whom I have the pleasure of knowing, are generally, intelligent, peaceable and courteous, having, as far as I can discover, the confidence of a very well-informed community, to which they are as much entitled as any equal number of medical gentlemen in any town that I visited. They have among them a chartered association styled the

### *Mobile Medical Society,*

which embraces the most popular practitioners of the city, and holds monthly meetings, at which papers are read and discussed. The society has also established a tariff of charges, by which a physician is permitted or required to charge three dollars per single visit, and five for two visits on the same day, furnishing his own medicines and charging for the same. I fear this information will set the mouths of some of our brethren on the banks of the Ohio, Green River, and the Wabash to watering; but a traveler, you know, is bound to tell the truth. The society is authorized by its charter to appoint out of its own body an

*Examining and Licensing Board,*

of five members, for the county of Mobile; and no scientific physician, even, I believe, a graduate, not thus licensed, can have the benefit of the courts of justice to collect his debts. Steam doctors, however, are exempt from this penalty! Verily, the legislature of Alabama must know much more of the science of cotton-planting than of medicine. I would advise them to look a little to consistency, and create a board of examiners, composed of men who can read, to ascertain that the disciples of that good, old mystical empiric, Samuel Thomson, really understand his book; without which they should not be allowed the benefit of the law. This measure, would, I fancy, soon drive them back to their *lasts* and *geese.*

I must, however, do the legislature the justice to state, that notwithstanding their tender admiration for the steamers, their respect for public opinion has deterred them from consituting that fraternity, the legal advisers of the city council; for, by its charter, the society is required, under the ordinances of the city, to organize a

*Board of Health,*

and procure and communicate the information necessary to the prevention of disease. To this end, the president, vice president, and secretary of the society constitute the advisory and executive cabinet of the council.

*The City Hospital.*

This is an extensive and commodious edifice, erected several years ago, so far from the Bay that it is still in the edge of town. It was designed strictly for the sick, and not as a receptacle for healthy paupers. Till last spring, it had the care of sick marines, and received from the general government the port money. The mass of its expenses was, however, defrayed out of the city treasury. At present it is rented to two enterprising physicians, Drs. Crawford and Ross, who, for a specific sum engage to receive, and take care of a given number of patients. The edifice is clean, well ventilated and appears to be managed in a faithful and efficient manner. A majority of the patients are Irish. There are wards appropriated for paying patients, and gentlemen who may be taken ill in Mobile, would do well to repair thither.

*Commercial Hospital.*

This was lately finished by the general government. It stands hard by the other, which, in architectural appearance, it rather overshadows. The plan is good, and in passing through it, I could not but be struck with the greater care bestowed by the Federal government on the wants of the water-men of the coast, than of the interior, which has been petitioning, unsuccessfully, for the last ten years. The physician who has charge of this public charity, is Dr. Gale.

*Lunatic Asylum.*

Neither Mobile nor the State of Alabama, has a hospital for the insane. It would be superfluous to say, that one is greatly needed. Eighteen months ago a gentleman was carried by his friends from the interior of this State to Ohio, and had to be brought back, because he could not for want of room be admitted into the excellent asylum at Columbus. Why do not the respectable and influential physicians of this State bring the subject before its general assembly; and continue pressing it on that honorable body, till an appropriation for a State institution be made? But it should not be established in Mobile, on account of the distance from the northern boundary of the State, and because its inmates might be invaded by yellow fever. Moreover, if it be erected at the seat of government, it will be more likely to receive the fostering care of the legislature, than if at a distance, where its touching scenes and precious blessings, would not display themselves to those who are to vote the annual supplies. I do not hesitate to predict, that if this subject should be forcibly, that is fairly, presented to the general assembly, the second session after such a presentation would afford an appropriation with which to commence an edifice, equal to the wants, and worthy of the character of this respectable State.

*School for the Blind and Deaf.*

These, although less urgently needed than a hospital for the insane, are important desiderata; and here, again, the children of misfortune and penury look up to the members of the medical profession. It is for them to make known to governors and legislators the resources of science and humanity, under the loss of sight and hearing; to plead for those whom they cannot restore; and establish themselves in the hearts of their fellow-citizens by proving themselves the benefactors of the unfortunate and obscure—for whom, too often, no body cares, because no body sets forth their infirmities, or points out the means of relief. The blind and deaf children of the rich may be sent away to the schools of another State, but those of poor parents necessarily grow up in ignorance. All such should be regarded as the children of the State, and be educated by her;—policy humanity and justice, alike, call for this public consideration. I say justice, because the State is endowed by the general government with a large landed income for the promotion of learning, in which the blind and deaf, without appropriate schools, can in nowise participate. But I have made my homily quite as long as becomes a stranger; and will break off, for the purpose of telling you something about

*Yellow Fever.*

Under the old Spanish and French regime, when Mobile was a village without wharves or ships, save an occasional lugger, when new comers were seldom seen in the crooked and narrow streets, and the people preferred fiddling and dancing to clearing off the drift and filth, which lodged in the margin of the stream, yellow fever never made its appearance. The first epidemic of which I am able to collect any imformation, was in 1819, since which it has recurred in 1821, '25, '27, '29, '37, '39, '42 and 1843, appearing sporadically in many other years. Of all these epidemics, I have by the kindness of the physicians and other gentlemen, collected a considerable number of facts. In reference to their origin, there is no diversity of opinion. I have not yet met with the first believer in importation. However they may disagree as to the mode of domestic origin, all concur in it as a reality. In reference to importation, Mobile is peculiarly situated, as the waters of the Bay are too shallow to admit of the approach of ships to the city, all of which, at least all the larger, lie 20 or 30 miles below, just within the Bay. I am far from considering the unanimity of opinion on this subject as conclusive; but it must be admitted to have in it the value of a fact; and I cannot but regard it is remarkable that, with an epidemic recurrence, on an average, every three years, if it were imported by ships which cast anchor at such distance from the city, its introduction from them should never have been detected.

*Dr. Dewees' Residence.*

Many of our readers have the name of Dewees so intimately associated with Philadelphia, as the theatre of those labors which finally made him the first man of America, in his line, that they may be surprised at the head line of this paragraph. In writing it I feel that I am but discharging a filial duty, for I was his pupil through the winter of 1805–6. It will be recollected, that after an apoplectic attack a few years before his death, he emigrated to this city, in the hope that its mild climate might renovate his crippled brain and nervous system. He immediately became the consulting physician of the place, and patients were brought to him from the interior, from Pensacola, and even New Orleans. Change of scene and climate, with these gratifying manifestations of confidence in his skill renovated his hopes, but failed to restore either his former health or mental power. While thus buoyed up, he engaged in building a large and curiously planned house, high up Government Street; and the pupil, who like myself, may make a pilgrimage to it, will find the unfinished, and dilapidated edifice inhabited by four poor families, with a school of eight little girls.

*Meteorological Observations.*

Mobile has a meteorologist, Dr. Stephen North, who makes observations worthy of being recorded, because confidence may be placed in their accuracy. This I am sorry to say is not always the case. I take pleasure in making him still better known, as it may lead observers in other places to seek exchanges of results with him, while this brief notice, is but an appropriate tribute of respect, for his patient fidelity as an observer.

*Plan of the City and its Environs.*

Mr. Lewis Troost, civil engineer and city surveyor, has just finished for me a map, of octavo size, which will show all the relations of water, dry land, and swamp, to the city. This will be one of a series, illustrative of the medical topography of the regions I have been, and still am exploring. I hope they will be found interesting and instructive.

*Smoking and Drinking.*

If one may judge from the number and populousness of the saloons and dram-shops, the number of drinkers, in this city, in proportion to its population, is not surpassed by any other city in the world. The temperance reform, in its palmiest days, seems to have extended less influence here than in most other places. The Irish laborers, who are very numerous, drink from instinct, and to support their national character, as it was before their emigration; but the saloons are as crowded as the doggeries. I am assured that not long since, there was a coffee house here, which cleared annually more than the salary of the President of the United States; and, at this time, the bar-room of the largest hotel of the city, is said to meet the expenses of the entire establishment. A large proportion of these customers are from those States in which public opinion has thrown its salutary restraints around young men, who, on the distant shores of the gulf, are indemnifying themselves for the deprivations under which they languished on the banks of the Delaware and the Hudson. Thus it is, that they prepare themselves for yellow fever; which, last year made its *debut* among a party of this kind, immediately after they had spent three days in a carousal over the Bay.

*Moral and Refined Society.*

Of this I feel abundant evidence every day, and its hospitality is equal to its intelligence and purity. Under this conviction, having finished my inquiries into its diseases, I shall leave here to-morrow for New Orleans, where your will hear from me again in due time.

